# Transient osteoporosis of the hip and subclinical hypothyroidism: an unusual dangerous duet? Case report and pathogenetic hypothesis

**DOI:** 10.1186/s12891-020-03574-x

**Published:** 2020-08-13

**Authors:** Marco Paoletta, Antimo Moretti, Sara Liguori, Matteo Bertone, Giuseppe Toro, Giovanni Iolascon

**Affiliations:** Department of Medical and Surgical Specialties and Dentistry, University of Campania “Luigi Vanvitelli”, Via De Crecchio 4, 80138 Naples, Italy

**Keywords:** Case report, Transient osteoporosis of the hip, Hypothyroidism, Diphosphonates, Rehabilitation

## Abstract

**Background:**

Transient osteoporosis of the hip (TOH) is a rare and temporary clinical condition characterised by bone marrow edema (BME), severe pain, and functional limitation. It commonly occurs in middle-aged men or in women in the last trimester of pregnancy. TOH usually resolves with conservative therapy but may predispose to hip fracture or progression to avascular necrosis (AVN). Etiology is still unclear, although several pathophysiological mechanisms underpinning this condition has been proposed. We describe the management of an unusual case of TOH occurred in a patient with subclinical hypothyroidism.

**Case presentation:**

A clinical case of a 46-year-old man with severe pain in the left anterior thigh is presented. After a comprehensive clinical and radiological approach, a TOH was diagnosed. Moreover, biochemical assessment suggested the presence of subclinical hypothyroidism. After 3 months of treatment with clodronate, physical therapy and hormone replacement therapy (HRT) a significant improvement of clinical and radiological outcomes was observed.

**Conclusion:**

Several pathological conditions have been related to development of TOH. In our case, we suggested for the first time a role of subclinical hypothyroidism as novel contributory factor for the onset of this condition, providing pathophysiological mechanisms and a scientific rationale for pharmacological treatment.

## Background

Transient osteoporosis (TO) is a rare disease characterized by bone edema as main finding, so that this condition is included among the bone marrow edema syndromes (BMES) [1]. In clinical practice, BMES are commonly underestimated and referred to interchangeably as bone marrow lesions (BMLs) that are characterized by progressive musculoskeletal pain with insidious onset, usually affecting a single joint, and functional impairment with limitations of activities of daily living (ADLs) [1]. The term “BMLs” defines conditions characterized by high bone marrow signal intensity on fluid-sensitive sequences on magnetic resonance imaging (MRI) [2]. This finding can be found in several traumatic, degenerative, inflammatory, vascular, metabolic, neoplastic and iatrogenic disorders [3].

Among BMLs, TO is characterized by low bone mineral density (BMD) affecting one skeletal site (such as hip or knee) and sometimes other bones [3–6]. Transient osteoporosis of the hip (TOH) is the most prevalent TO, mainly affecting men in middle age, even if pregnancy is considered as the most common risk factor [7], as well as the first cause of TOH described in the literature [8]. However, it is essential to identify primary (idiopathic) or secondary forms of TOH [7], where radiological finding of BMLs could be suggestive of systemic conditions.

In this case report we describe an unusual form of TOH related to subclinical hypothyroidism describing clinical and diagnostic work-up as well as therapeutic management and proposing a pathophysiological hypothesis.

## Case presentation

A 46-year-old Caucasian man referred to our outpatient rehabilitation service in January 2019 for spontaneous pain at the left thigh. He was 1.80 m tall and weighed 72 kg (BMI 22.2 kg/m^2^). He reports a lifestyle characterized by occasional alcohol intake (less than one alcohol unit per day) and sedentary work (office worker). Medical history is significant for a trimalleolar fracture of the right ankle treated with open reduction and internal fixation in October 2011. Moreover, in July 2018 he practiced stool culture, faecal occult blood test, celiac disease screening, urinalysis, and abdominal ultrasound, because of the occurrence of several episodes of acute diarrhoea, that were responsive to a short course of antidiarrheal and antispasmodic agents and probiotics.

Clinical complaints started in January 2019 with gradually increasing groin pain without any previous trauma requiring the use of a crutch. The primary care physician prescribed oral diclofenac 150 mg and omeprazole 20 mg per day for 1 week, but the symptoms did not regress. Therefore, his doctor advised him to consult a physiatrist at our service.

**Fig. 1:**
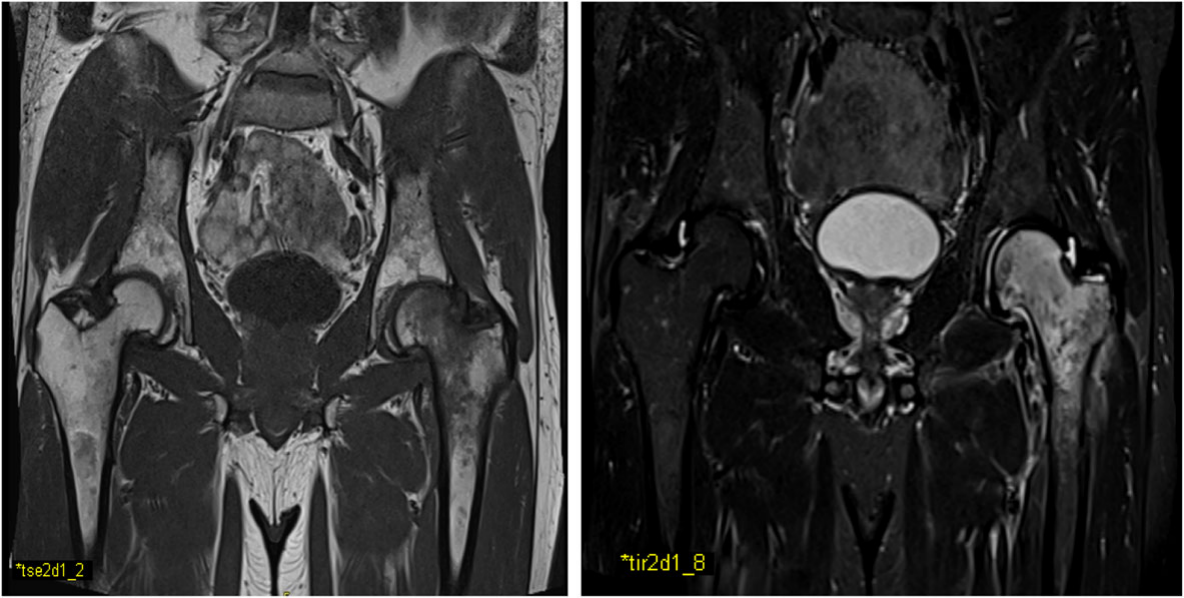
Left lower limb MRI showing remarkable and diffuse bone marrow edema

On physical examination, patient reported severe groin pain (Numeric Rating Scale, NRS, 8/10) radiating to the antero-medial thigh and at the knee, worse during the night and weight-bearing activity. Moreover, passive and active range of motion (ROM) of the left hip (internal and external rotation 15°, flexion 95°) were limited and patients was forced to use walking sticks. We planned a diagnostic workup including laboratory exams, including complete blood count (CBC), serum erythrocyte sedimentation rate (ESR), C-Reactive Protein (CRP), alanine transaminase (ALT), aspartate transaminase (AST), creatinine, uric acid, alkaline phosphatase (ALP), calcium, phosphate, parathyroid hormone (PTH), 25(OH) vitamin D, thyroid-stimulating hormone (TSH), total testosterone, protein electrophoresis, urinary free kappa and lambda light chains, and magnetic resonance imaging (MRI) scan of the hips. Laboratory tests were normal, except for increased serum TSH (3.28 μIU/ml, normal range 0.2–2.5 μΙU/ml), while remarkable and diffuse bone edema in epiphyseal and metaphyseal region of the left proximal femur was reported, supporting the diagnosis of TOH (Fig. 1).

Therefore, we prescribed clodronate (200 mg i.m. for 10 days and then 200 mg i.m. every other day for 20 days) [9], oral calcium citrate (1 stick of 500 mg per day for 1 month), and cholecalciferol (1 oral solution of 25,000 IU weekly for 1 month). Pharmacological approach was associated with instrumental physical therapy, including Pulsed Electromagnetic Fields (PEMFs) stimulation [8 h per day (night use) for 6 weeks; the device generated single-voltage pulses of 1.3 milliseconds in duration, with a frequency of 75 Hz, and was positioned on the lateral thigh] [10–12], and Neuromuscular Electrical Stimulation (NMES) [13] of the left quadriceps (1 session per day for 3 weeks; electrodes were placed around the thigh for 30 min each session, generating a frequency of 50 Hz, pulse duration of 250 ms, and 10 s on and 30 s off). Moreover, protected weight bearing for 3 weeks was advised. Finally, we recommended a consultation with an endocrinologist to address putative thyroid disorders.

The endocrinologist performed thyroid ultrasound revealing hypoechoic nodule of 5 mm and new laboratory assessment confirming increased TSH levels (3.67 μΙU/ml) and normal level of free thyroxine, (FT4, 15.18 pg/ml, normal range 6–18 pg/ml) and free triiodothyronine, (T3, 3.67 pg/ml, normal range 2.57–4.43 pg/ml). The specialist made the diagnosis of subclinical hypothyroidism and prescribed a nutraceutical containing both myo-inositol (600 mg) and selenium (83 mcg) (1 tablet per day for 2 months).

**Fig. 2:**
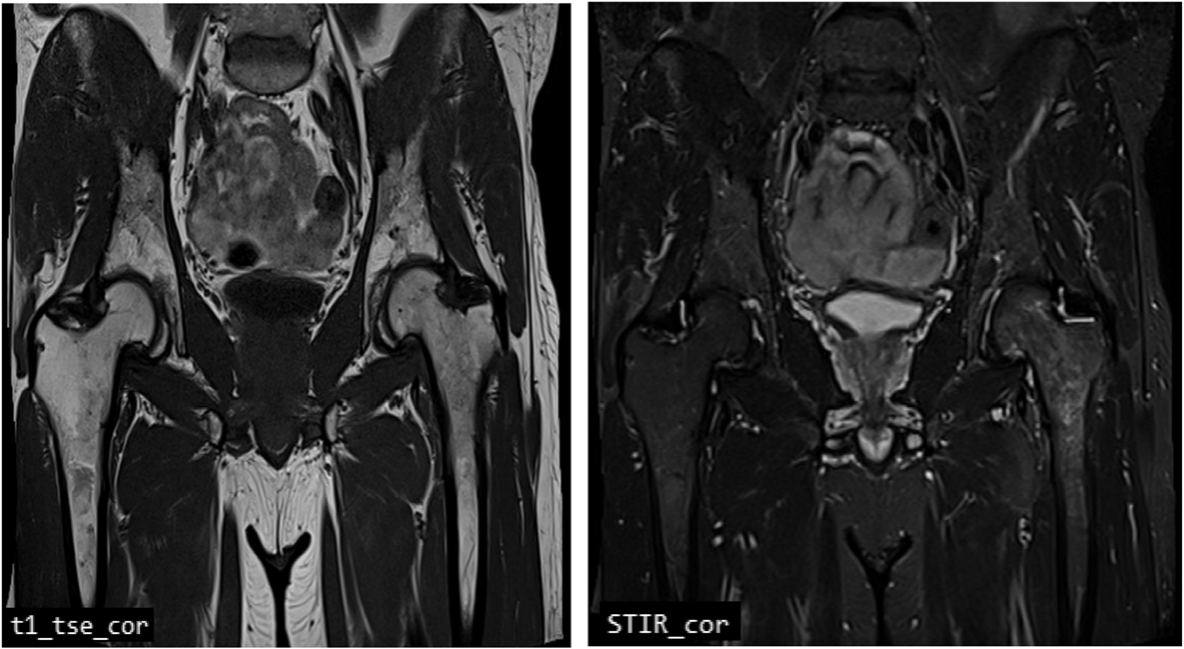
Left hip MRI showing a significant reduction of bone edema at proximal femur

Clinical and instrumental follow-up performed after 2 months from the beginning of therapies, showed significant pain relief (NRS 2/10), improved ROM of the hip, and a significant reduction of bone edema at MRI examination of the left hip (Fig. 2). Moreover, the patient was able to walk without aids. However, due to persistent high level of TSH (3,56 μΙU/ml, normal range 0,2–2,5 μΙU/ml), endocrinologist prescribed levothyroxine 25 μg per day resulting in serum TSH reduction (2,64 μΙU/ml) after 2 months. Finally, the patient did not report any adverse events.

## Discussion and conclusion

Bone marrow lesions (BMLs) are characterized by high signal on both T2/proton density with fat suppression and short tau inversion recovery (STIR) MRI sequences with or without low signal intensity on T1-weighted images [14]. These conditions are generally defined as bone marrow edema (BME), a nonspecific MRI finding in both symptomatic and asymptomatic patients. Transient osteoporosis of the hip is included in non-traumatic BMES [14], whose pathogenic mechanisms are not well defined. Our patient is an office worker conducting a sedentary lifestyle, thus suggesting a non-traumatic trigger of TOH. BMES is usually burdened by a challenging differential diagnosis [14–16], considering that trauma, infection, inflammation, degenerative process, ischemic injury, neoplasia, surgery, drugs, neurologic or metabolic disorders [7] might be associated to its occurrence. Moreover, all these conditions might contribute to impaired bone metabolism. Particularly, severe hypothyroidism negatively influences bone modeling and skeletal growth in children, whereas in adults it leads to delayed remodeling of cortical and trabecular bone because of abnormal osteoblastic and osteoclastic activity [17–20].

Some cases of TOH due to severe hypothyroidism have been reported in the literature. In 1938 Albright first described radiological lesions of hip simulating Legg-Perthes disease, reversible after hormone replacement therapy (HRT) in a 13-year-old hypothyroid child with growth retardation [21]. In 1959 Weissbein et al. described a case of a 22-year-old man affected by primary myxedema that referred to orthopedic service for severe pain of anterior region of the thigh to the knee aggravated by walking. Plain x-ray demonstrated an osteolytic lesion of the right femoral head improved after HRT [22]. More recently, McLean and Podel reported a case of a 25-year-old man with severe hypothyroidism and right hip and knee pain on weight-bearing and effusion of the right hip at MRI evaluation, improving within 2 to 3 months of HRT [17]. About 15 years later, Mepani and Findling described a case of a 32-years-old man with severe primary hypothyroidism, with 3-to-4 months hip discomfort and BME of the femoral head at MRI improving at 3 months and 1 year after HRT [23].

However, the cases mentioned share the occurrence of spontaneous bone pain due to lesions at femoral head in patients with severe hypothyroidism.

On the other hand, our case is unique because the occurrence of TOH in a patient with subclinical hypothyroidism has not previously been described in the literature so far. We speculate that subclinical hypothyroidism may be a metabolic trigger for TOH as reported for severe hypothyroidism [17, 18], resulting in fatty replacement of bone marrow [24], cellular infiltration (lymphocytes, plasma cells, histiocyte) [25], impaired blood flow (impaired venous return), local hyperemia [26–29], and pro-inflammatory milieu (cytokines) [3]. These pathways may cause a vicious circle with an increased bone turnover and acceleration of biological processes called Regional Acceleratory Phenomen (RAP) [30, 31]. The rationale for using antiresorptive drugs like bisphosphonates (BPs) could be their action on increased regional high bone turnover state, pro-inflammatory milieu (cytokines) and vasoactive agents, resulting in clinical and radiological improvements [3].

The role of BPs in relieving bone pain has been extensively hypothesized. Osteoclastic activity and/or bone inflammation produce an acidic microenvironment that activates specific chemoreceptors (TRVP1 and ASICs) that may be involved in the pathogenesis of pain [32, 33]. Therefore, by inhibiting osteoclast activity BPs might be effective in reducing bone pain. Moreover, in rat models, alendronate raises pain threshold, reducing the number of c-Fos + neurons (proto-oncogene expressed by neurons following both nociceptive and non-nociceptive stimuli) in lamina 1 and 2 of the dorsal horns of the spinal cord [34]. Also, the release of substance P and other neuropeptides may be implicated in mechanisms of bone pain. In a rat model of sciatic nerve injury, it has been hypothesized that substance P may be implicated even in BMD changes [35]. Bisphosphonates may reduce levels of substance P via the TNF-α pathway playing a key role in inflammatory pain transmission by primary sensory nerves [36–38]. Among BPs, clodronate seems to have peculiar anti-inflammatory activity thanks to its intracellular metabolites (such as AppCCl_2_p produced by RAW 264 macrophages) acting on cytokines and NO release reducing DNA binding activity of NF-kB [39].

Alongside anti-resorptive and anti-inflammatory action, analgesic mechanisms of clodronate have been proposed. This non-nitrogen-containing BPs (non-N-BPs) blocks phosphate transporter family SLC17 and inhibits vesicular transporters of ATP and/or glutamate [40]. Hydrolyzed products of ATP and ATP released in extracellular environment stimulate purinoceptors (P2X or P2Y and P1 adenosine receptor) on peripheral sensory nerves involved in pain transmission and modulation. Clodronate is a presynaptic blocker of vesicular ATP release from neurons that might decrease neuropathic and inflammatory pain by slowing down purinergic chemical transmission [41].

On the other hand, anti-resorptive effects of clodronate depends by intracellular accumulation of toxic ATP analogs [42, 43] and inhibition of mitochondrial ATP translocases [44] in osteoclasts resulting in their apoptosis.

Anti-resorptive, anti-inflammatory and analgesic properties of clodronate make it a viable strategy to manage BMES and several studies have evaluated its efficacy in these conditions. In two RCTs, daily intravenous clodronate (300 mg daily for 10 or 12 consecutive days) has been investigated to treat algodystrophy with clinical recovery in 1–2 months [38]. The same dosing regimen was used in three patients with TOH followed by physical therapy (3 weeks of flexibility exercises) with clinical recovery and BMD improvement at 3–4 months [45]. Similar results were reported in a 30-year-old woman with TOH using clodronate in association with calcium and vitamin D supplementation after 2 months [46].

Even if this therapeutic protocol seems to be commonly suggested for BMES [7], recently, some authors assert that a global dose of 3000 mg of clodronate would appear insufficient [9]. In our case we used for the first time intramuscular clodronate for the management of TOH. Our protocol consists of intramuscular clodronate at the dose of 200 mg daily for 10 consecutive days and followed by 200 mg every other day for 20 days reaching a total dose of 4000 mg, as proposed by Frediani et al. [9, 47].

However, clodronate and other BPs must be carefully administered in patients with low serum ALP, commonly noticed in patients with severe hypothyroidism [18], because of higher risk of atypical femoral fractures as reported for patients with hypophosphatasia [48]. Our patient had normal serum serum ALP (68.3 U/l, normal range 40–129 U/l) that allows to administer BPs safely.

We combined instrumental physical therapy with pharmacological treatment for the first time in the management of TOH. It has been hypothesized that PEMFs may preserve subchondral bone from marrow edema and stimulate osteogenic activity reducing the risk of trabecular fracture in femoral head osteonecrosis [12]. Moreover, PEMFs may promote bone formation, antioxidant and adenosine receptors synthesis reducing pro-inflammatory cytokines in CRPS I [49]. Concerning NMES, encouraging results were also reported in reducing pain after 6 weeks in patients with osteonecrosis of the femoral head [13].

Based on the available evidence, we assumed that combining PEMFs, NMES and clodronate may have been a suitable treatment strategy for our case of TOH.

Thyroid HRT would seem to be an effective approach in patient with TOH and severe hypothyroidism [17, 21–23]. However, in our case we used promptly a higher dose of clodronate (4000 mg in 1 month). Only belatedly endocrinologist added levothyroxine, when bone edema already seemed in resolution.

We postulate that a multimodal intervention based on the use of clodronate and physical therapy could improve the clinical outcome in patients with TOH caused by subclinical hypothyroidism. In conclusion, TOH is suggestive of abnormal bone metabolism. Diagnostic process has a pivotal role for revealing secondary forms of TOH. Starting an appropriate and timely intervention is mandatory for a complete recovery from TOH to avoid the progression towards avascular necrosis of the hip.

## Data Availability

The data that support the findings of this study are available from the corresponding author upon reasonable request.

## Abbreviations

TO: Transient osteoporosis
TOH: Transient osteoporosis of the hip
BME: Bone marrow edema
AVN: Avascular necrosis
HRT: Hormone replacement therapy
BMES: Bone marrow edema syndromes
BML: Bone marrow lesion
ADL: Activities of daily living
BMI: Body mass index
MRI: Magnetic resonance imaging
BMD: Bone mineral density
NRS: Numeric Rating Scale
ROM: Range of motion
CBC: Complete blood count
ESR: Erythrocyte sedimentation rate
CRP: C-Reactive Protein
ALT: Alanine transaminase
AST: Aspartate transaminase
ALP: Alkaline phosphatase
PTH: Parathyroid hormone
TSH: Thyroid-stimulating hormone
IU: International unit
i.m.: Intramuscular injection
PEMF: Pulsed Electromagnetic Field
NMES: Neuromuscular Electrical Stimulation
STIR: Short tau inversion recovery
RAP: Regional Acceleratory Phenomen
BPs: Bisphosphonates
TRVP1: Transient receptor potential cation channel subfamily V member 1
ASICs: Acid-sensing ion channels
AppCCl2p: Adenosine 5′-(beta,gamma-dichloromethylene) triphosphate
TNF-α: Tumor necrosis factor-α
NO: Nitric oxide
NF-kB: Nuclear factor kappa-light-chain-enhancer of activated B cells
ATP: Adenosine triphosphate
SLC: Solute carrier family 17
RCT: Randomized controlled trial
